# Opportunistic Pathogens and Microbial Communities and Their Associations with Sediment Physical Parameters in Drinking Water Storage Tank Sediments

**DOI:** 10.3390/pathogens6040054

**Published:** 2017-10-26

**Authors:** Ke Qin, Ian Struewing, Jorge Santo Domingo, Darren Lytle, Jingrang Lu

**Affiliations:** 1 ORISE, Office of Research and Development, U. S. Environmental Protection Agency, Cincinnati, OH 45268, USA; qin.ke@epa.gov; 2 Pegasus Service Inc., Cincinnati, OH 45268, USA; struewing.ian@epa.gov; 3 Office of Research and Development, U. S. Environmental Protection Agency, Cincinnati, OH 45268, USA; Santodomingo.Jorge@epa.gov (J.S.D.); lytle.darren@epa.gov (D.L.)

**Keywords:** *Legionella*, opportunistic pathogen, storage tank sediment, microbial community, element, corrosion

## Abstract

The occurrence and densities of opportunistic pathogens (OPs), the microbial community structure, and their associations with sediment elements from eight water storage tanks in Ohio, West Virginia, and Texas were investigated. The elemental composition of sediments was measured through X-ray fluorescence (XRF) spectra. The occurrence and densities of OPs and amoeba hosts (i.e., *Legionella* spp. and *L*. *pneumophila*, *Mycobacterium* spp., *P. aeruginosa*, *V. vermiformis, Acanthamoeba* spp.) were determined using genus- or species-specific qPCR assays. Microbial community analysis was performed using next generation sequencing on the Illumina Miseq platform. *Mycobacterium* spp. were most frequently detected in the sediments and water samples (88% and 88%), followed by *Legionella* spp. (50% and 50%), *Acanthamoeba* spp. (63% and 13%), *V. vermiformis* (50% and 25%), and *P. aeruginosa* (0 and 50%) by qPCR method. *Comamonadaceae* (22.8%), *Sphingomonadaceae* (10.3%), and *Oxalobacteraceae* (10.1%) were the most dominant families by sequencing method. Microbial communities in water samples were mostly separated with those in sediment samples, suggesting differences of communities between two matrices even in the same location. There were associations of OPs with microbial communities. Both OPs and microbial community structures were positively associated with some elements (Al and K) in sediments mainly from pipe material corrosions. Opportunistic pathogens presented in both water and sediments, and the latter could act as a reservoir of microbial contamination. There appears to be an association between potential opportunistic pathogens and microbial community structures. These microbial communities may be influenced by constituents within storage tank sediments. The results imply that compositions of microbial community and elements may influence and indicate microbial water quality and pipeline corrosion, and that these constituents may be important for optimal storage tank management within a distribution system.

## 1. Introduction

Drinking water distribution systems (DWDS) contain complex microbial communities based on both measured by composition [1,2] and genetic network [3]. While drinking water treatment and disinfection considerably reduces risk of exposure, opportunistic pathogens (OP) are often detected in different parts of DWDS. Although most of the attention has been given to the microbiology of drinking water in premise plumbing and biofilms on pipes, drinking water storage tank sediment has also been shown to harbor OPs [4,5]. Many of these OP, such as *Legionella* spp., *Mycobacterium* spp., *Pseudomonas aeruginosa*, and some of *Acanthamoeba* spp. can pose significant risks to immunocompromised people [6,7,8,9,10]. Free-living amoeba (FLA), such as *Vermamoeba vermiformis*, and some groups of *Acanthamoeba* spp. are also relevant to public health as they are potential hosts to several OPs [7].

Microbial growth within DWDS biofilms has been associated with corrosion of pipes, and the production of various elements in water and sediments in DWDS [11,12,13]. Teng et al. (2008) found that biofilm can greatly affect element composition and the crystalline phase of corrosion scales. Also, studies have shown that iron-oxidizing bacteria can accelerate corrosion early on, while iron-reducing bacteria (IRB) may inhibit corrosion in later stages, processes that may be influenced by changes in biofilm microbial diversity [12]. DWDS sediments are mainly composed of iron, sulfur, total organic carbon (TOC), calcium, total inorganic carbon (TIC), phosphorous, manganese, magnesium, aluminum, and zinc, and can concentrate trace regulated contaminants such as arsenic and radium [14,15,16]. The properties distribution system sediment such as elemental composition may impact the composition and structure of their microbial communities. Stein et al. (2001), for example, found that iron- and manganese-enriched sediments also contained two groups related to known metal-oxidizing genera, Leptothrix of the β-Proteobacteria and Hyphomicrobium of the α-Proteobacteria, and a Fe(III)-reducing group related to the Magnetospirillum genus of the α-Proteobacteria [17]. White et al. (2011) reported on the microbiological community structure of sediment and corrosion by-products associated with a DWDS lead pipe using rRNA gene sequencing. They identified bacteria species that have previously reported in heavy-metal-contaminated soils that could potentially impact metal mobility [18].

Elements associated with corrosion by-products can also have an impact in the survival of OPs [19,20,21,22]. If elements can protect and promote growth of OPs in storage tank sediments, they may potentially be released into DWDS [23]. Microbial communities could also be impacted by elemental composition of sediments and the redox condition [24,25,26]. However, relatively little is known on the microbial community in sediments in drinking water storage tanks due to sampling, because samples can only be obtained randomly from tank clean companies [2]. To address this, we examined the occurrence and densities of OPs, the microbial community structure, and their associations with sediment elements. We detected OPs using qPCR [4] and determined the microbial community compositions using next generation sequencing.

## 2. Materials and Methods

### 2.1. Sample Collection

Water and sediment samples were collected while industrial storage & water tank cleaning companies were conducting tank cleaning. The samples were collected from eight water storage tanks (16 sediments and water samples) in Ohio (D39-42), West Virginia (D43), and Texas (D50, D53 and D54). For microbial community analysis, an additional sample from Ohio was processed (D84, sediment samples only, collected in triplicate). Sediment and water samples were transferred to sterile containers using aseptic techniques. Collected samples were then placed into ice coolers and shipped overnight to the EPA laboratory (Cincinnati, OH, USA) for different analyses. Water from sediment samples was first removed via centrifugation at 2844× *g* for 3 min using a Swing Bucket Rotor on a Thermo Sorvall^®^ Legend^®^ T Plus centrifuge (Thermo Fisher Scientific., Waltham, MA, USA) and 0.25 g was used to extract DNA using PowerSoil^®^ DNA Isolation Kit (MoBio, Carlsbad, CA, USA) following the manufacturer’s instruction. Water samples (1 L) were filtered using EMD Millipore Durapore^TM^ membrane filter (0.40 µm, MilliPore, Foster City, CA, USA). The filtered biomass was lysed using a Beadbeater (BioSpec Products, Inc., Bartlesville, OK, USA) for 1 min. Tubes were centrifuged at 12,000× *g* for 5 min and the supernatant transferred to sterile microcentrifuge tubes. The DNA extraction was completed using the Master Pure Complete DNA and RNA Purification Kit (Epicentre Technologies Corp., Madison, WI, USA). Extracted DNA was resuspended in 50 µL of molecular grade water. DNA concentrations were measured using Nanodrop 2000 spectrophotometer (NanoDrop Technologies, Inc., Wilmington, DE, USA). Remaining sediment samples were air dried for a period of three days after which elemental composition, and their bulk and particle density were measured with graduated cylinder and mass balance. For the bulk density, about 2 to 3 g of the sample were weighed and the mass was recorded. Then, the sample was placed in a graduated cylinder and the volume was recorded. This volume was the bulk volume because it included the pore spaces between particles. The bulk density was then calculated by dividing the recorded mass by the recorded volume. The procedure was the same for the particle density except the particle volume was determined by the displacement of water.

Porosity was calculated by the following equation:

$$\mathrm{Porosity}=1-\frac{bulkdensity}{particledensity}$$

The elemental composition of the sediments was measured through X-ray fluorescence (XRF) of the pressed dried sediments pellets using a Panalytical Axios Instrument (Panalytical, Almelo, The Netherlands).

### 2.2. qPCR Analysis

The occurrence and densities of OPs and amoeba hosts (i.e., *Legionella* spp. and *L*. *pneumophila*, *Mycobacterium* spp., *P. aeruginosa*, *V. vermiformis, Acanthamoeba* spp.) were determined using genus- or species-specific qPCR assays, as previously described [2]. Briefly, qPCR assays were performed on a QuantStudio 6 Flex Real-Time PCR System (Applied Biosystems, Foster City, CA, USA) with reaction mixtures. To prevent carryover contamination, an initial incubation at 50 °C for 2 min with uracil-N-glycosylase (UNG) at the onset of the cycling program was conducted before the one cycle of denaturation and enzyme activation at 95 °C for 10 min. The following cycling conditions were used as 40 cycles at 95 °C for 15 s and at the specific Tm (°C) listed in Table S1 for 30 s with an extension at 72 °C for 30 s and a final hold at 72 °C for 5 min, with qPCR reactions for each DNA sample undertaken in duplicate. The standard curves were generated using genomic DNA from each of the targeted groups. Target cells in the extracts are reported as genome copy numbers (GN). The presence of potential qPCR inhibitors was determined using 10-fold dilution of each extract. DNA standards and no-template controls were included on each PCR run.

### 2.3. Next Generation Sequencing

Sequencing libraries were constructed using water and sediment DNA extracts as PCR templates coupled with barcoded primers targeting the V3–V4 region of the 16S rRNA gene [27]. The reactions used to generate the sequencing libraries were performed in 25 µL volumes using the Ex Taq kit (Takara) with 200 nM for each of the forward and reverse primer and 2 µL of nucleic acid template. Cycling conditions involved an initial 5 min denaturing step at 94 °C, followed by 30 cycles of 45 s at 94 °C, 60 s at 50 °C, and 90 s at 72 °C, and a final elongation step of 10 min at 72 °C. Agarose gel electrophoresis was used to confirm the size of the amplification products. PCR products were then pooled and size selected prior to multiplex sequencing on an Illumina MiSeq benchtop sequencer using paired-end 250 bp kits at the Cincinnati Children’s Hospital DNA Core facility. Sequence reads were processed using MOTHUR software [28] as described earlier [3]. Briefly, before analysis, sequence reads with overall low quality scores, or containing homopolymers (>8 nucleotides) and ambiguous base calls (N’s), were removed from further analysis [29]. MOTHUR aligned and sorted reads with >97% similarity into operational taxonomic units (OTUs). Chimeric OTUs were screened out by the UCHIME algorithm built within MOTHUR.

### 2.4. Data Analysis

Statistical analyses were performed using Rstudio (https://www.rstudio.com/). Paired *t*-test was performed to test if there was any significant difference between genome copy number of OP in sediment and water samples. Pearson’s linear correlation coefficients were calculated to test the correlation within OP and between OP and environmental physical parameters. For the microbial community analysis, one-way ANOVA was performed to test if there were significant differences between sediment and water samples. Nonmetric multidimensional scaling (NMDS, which acted to visualize the community structure and correlated physicochemical conditions using metaMDS function in the ‘vegan’ package) was used to cluster microbial communities based on the similarity of OTU distribution. Multiple response permutation procedure (mrpp) analysis was performed to test if there were significant differences in the NMDS plot between water and sediment samples (i.e., if there were two different groups). Correlation coefficients were calculated to check if there was any environmental factor that could explain clustering on the NMDS plot at a statistically significant level.

## 3. Results

### 3.1. Opportunistic Pathogens

*Mycobacterium* spp. were the most frequently detected OPs in the sediments and water samples (88% and 88%, respectively), followed by *Legionella* spp. (50% and 50%), *Acanthamoeba* spp. (63% and 13%), *V. vermiformis* (50% and 25%), and *P. aeruginosa* (0 and 50%). The occurrence of free living amoebae was higher in sediment than in water. *L. pneumophila* was also detected in both sediment (25%) and water (13%) (Table 1). *Mycobacterium* spp., *Legionella* spp., and FLA constituted major OPs, and there were significant correlations between *Legionella* spp. and *Mycobacterium* spp. The major OPs in the sediments presented the highest densities in Texas samples (D53 and D54), followed by Ohio samples (D39 and D40). However, in the water samples, the highest densities of the major OPs occurred in Ohio samples (D39 and D40), followed by the West Virginia sample (D43) and one Texas sample (D50). It should be noted that FLA and *L. pneumophila* occurred more frequently in sediments than in water; *P. aeruginosa* occurred more frequently in water than in sediments (Table 1).

The major elements measured in sediments were Zn (18.7%), Fe (16.0%), Si (7.1%), Mn (3.6%), Al (3.4%), and Ca (3.2%) (Table S2). Element densities were different among samples and locations. For example, particle densities, porosities, Ca, Mg, and Zn were higher in OH samples (D39–42) than in TX samples (D50–54) (Table S3). The Pearson’s linear correlation coefficients for each of the OPs studied with the sediment elements measured are listed in Table 2. It showed that OPs were associated with some sediment elements. Specifically, *V. vermiformis*, *Mycobacterium*, *Legionella*, and *L. pneumophila* were positively associated with Al and K. (Table 2). 

### 3.2. Microbial Community

**Figure 1:**
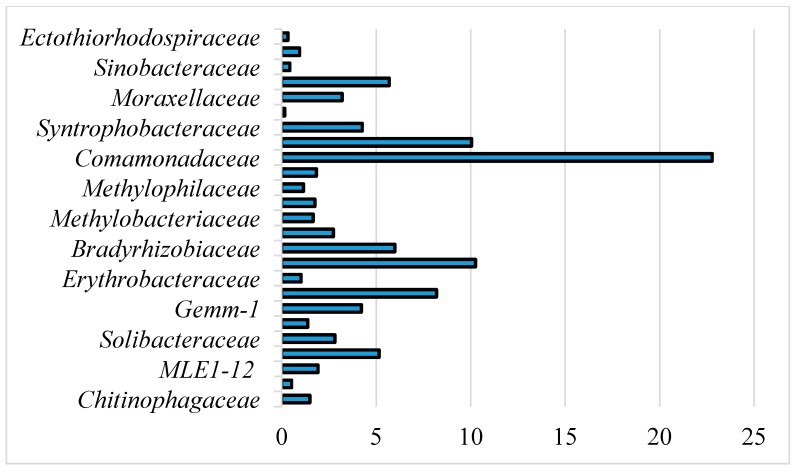
The 25 most abundant families presented in all the samples.

A total of 25 bacterial families were observed among the OTUs with relative abundance >1% (Figure 1). *Pseudomonadaceae* (19.4%) *Comamonadaceae* (18.5%), *Sinobacteraceae* (15.5%), and *Sphingomonadaceae* (13.0%) were the most dominant, but that of *Mycobacteriaceae* was less than 1%. There were associations of microbial communities between the sediment and water in the same site (Table S4). Many families like *Sphingomonadaceae* presented both in water and sediments in many sites (Table S4). Dominant OTUs varied with samples or locations. For OH samples, *Comamonadaceae* dominated in D39 of both sediments (48.9%) and water samples (90.6%), and water samples in D40 (42.8%) and D41 (38.9%). The dominance of *Comamonadaceae* in water and sediment samples has been previously documented [30,31], probably due to their abilities to restrain other competitors [32]. *Syntrophobacteraceae*, anaerobic sulfur-reducing bacteria [33] prevalent in sediments and soil [34,35], was most abundant in D40 sediments (28.0%). *Oxalobacteraceae*, a ubiquitous family found in drinking water systems [36], dominated in D41 sediment samples (47.5%) and D42 water samples (60.1%), while *Bradyrhizobiaceae* (18.3%) were most abundant in D42 sediment samples. For TX samples, *Pseudomonadaceae* (59.8%) dominated in D53 sediment samples, while *Oxalobacteraceae* (45.12%) represented the majority in water samples. The most abundant families in terms of OTUs percentage in D54 sediment and water samples were *Nitrospiraceae* (57.4%) and *Sphingomonadaceae* (50.2%), respectively.

**Figure 2:**
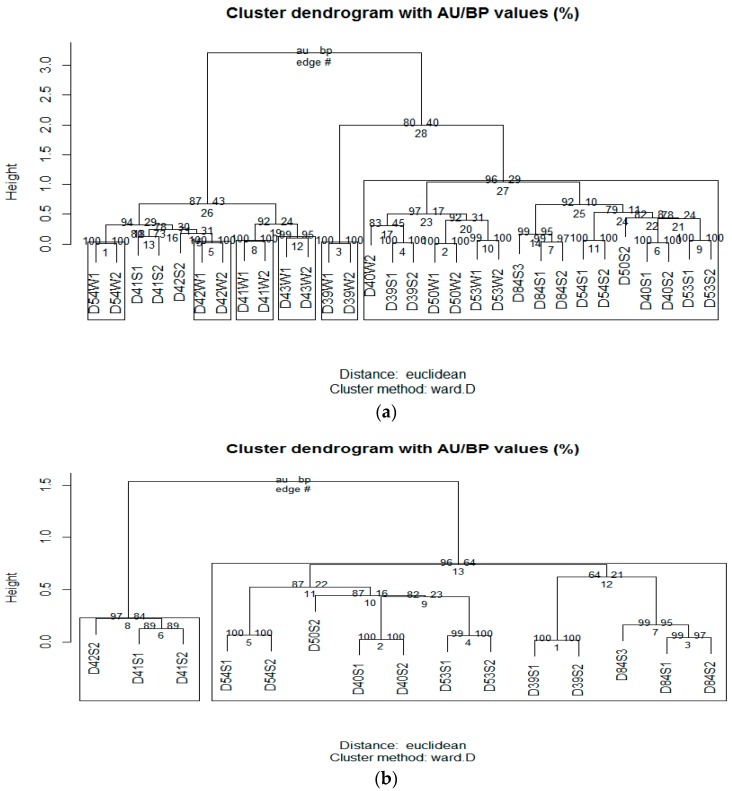
Cluster dendrogram of both sediment and water samples (**a**); and only sediment samples (**b**) based on microbial community structures. Values on the edges of the clustering are *p*-values (%). Top left values are approximately unbiased (AU) *p*-values, and top right values are bootstrap probability (BP) values. Clusters with AU larger than 95% are strongly supported by data.

The samples sequenced in both water and sediment were clustered into one major group and several smaller groups according to relative abundance of the 25 families (Figure 2a). The sample type (water and sediment) could be one of the factors explaining microbial community structure. All the sediment samples except for D41 and D42 were in the major group. In sediment samples, the family (*Solibacteraceae*) was significantly higher in sediment than water (6.2% vs. 0.2%, *p* = 0.02, D40, etc.). The location of water system could be another factor, as shown by the two distinct clusters. Two samples from one city (D41 and D42) were clustered together separately from other samples (Figure 2b). 

**Figure 3:**
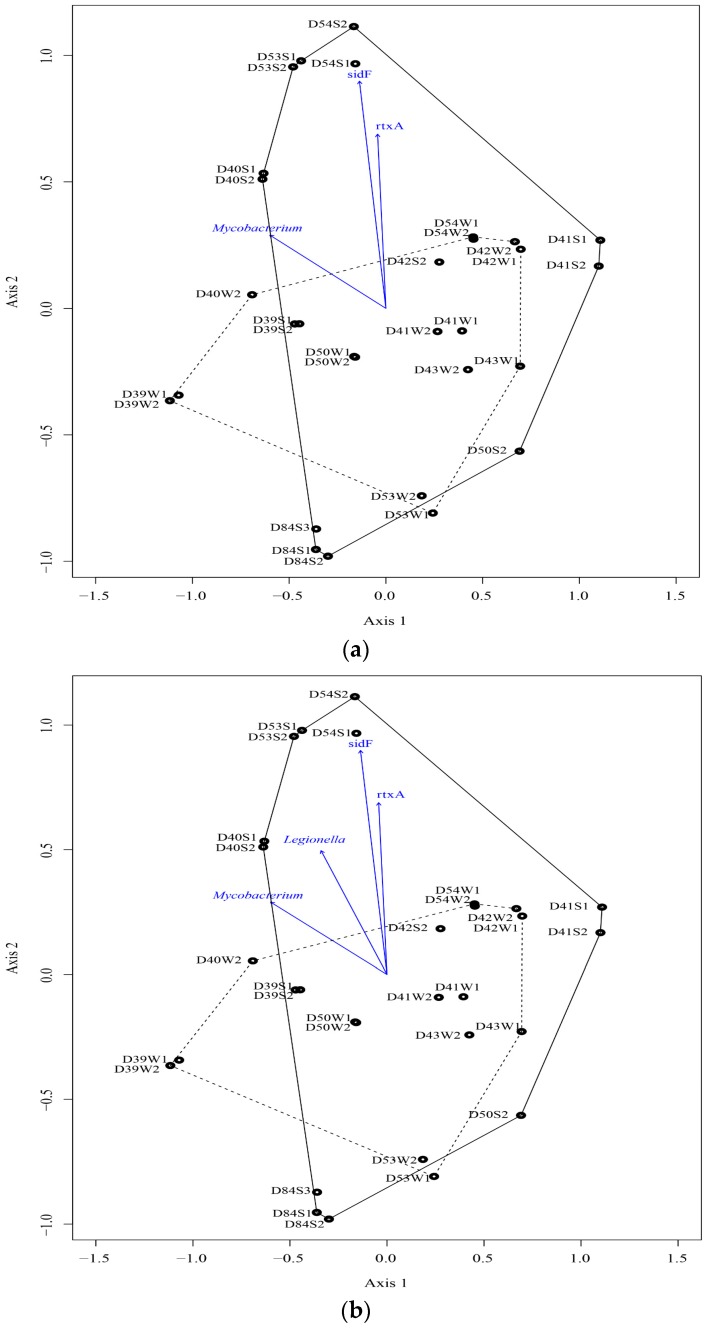
Nonmetric multidimensional scaling (NMDS) of paired water (W) and sediment (S) samples (Ohio: D39-42, West Virginia: D43, and Texas: D50, D53, and D54) based on microbial community structures with operational taxonomic unit (OTU) numbers and relative abundance of each OTU against relative abundance of elements and opportunistic pathogen densities by qPCR at significant level 10% (**a**) and 5% (**b**). The solid circles represent the central tendency of all OTUs detected in each sample from the sediment and water samples, respectively. Symbols that are closer to each other with a dash circle depict samples that contain similar taxa detected by the sequencing. A biplot is overlaid on the ordination to identify environmental parameters that were correlated with the microbial community structure. The length of the line corresponds to the degree of the correlation.

**Figure 4:**
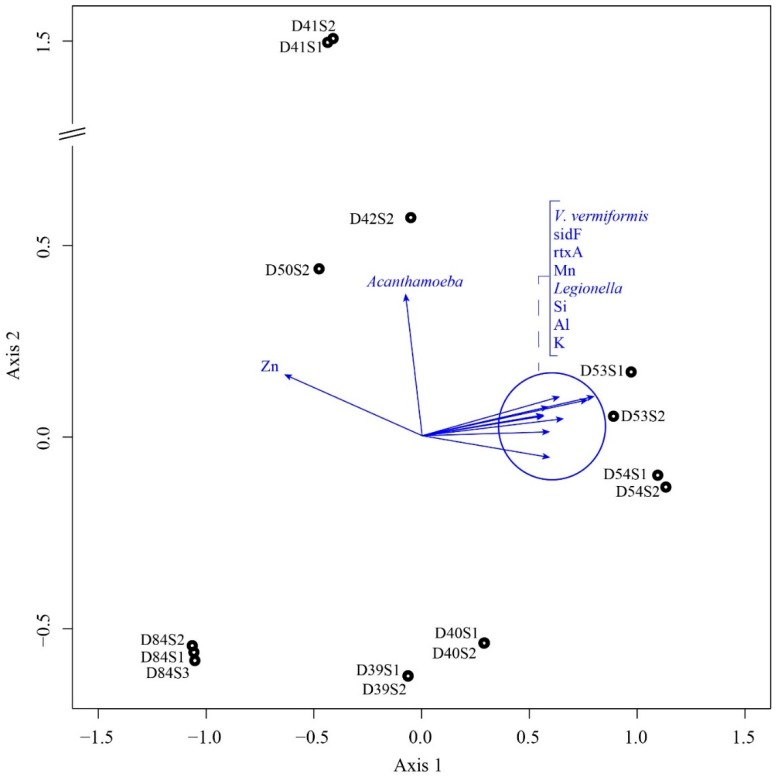
NMDS of sediment (S) samples (Ohio: D39-42, West Virginia: D43, and Texas: D50, D53, and D54) based on microbial community structures with OTU numbers and relative abundance of each OTU against relative abundance of elements and opportunistic pathogen densities by qPCR. The solid circles represent the central tendency of all OTUs detected in each sample from the sediment and water samples, respectively. Symbols that are closer to each other with a dash circle depict samples that contain similar taxa detected by the sequencing. A biplot is overlaid on the ordination to identify environmental parameters that were correlated with the microbial community structure. The length of the line corresponds to the degree of the correlation. Only variables that had a significant correlation (*p* < 0.05) are depicted.

When considering the element’s impact on community structure, most microbial communities in water samples (circled with a dash line) were separated from those in sediment samples (with a solid line) (Figure 3a), as inferred from the analysis of NMDS. Only the communities in D39 and D42 water and sediment, which were positioned within the overlap of the two circled areas, were not separated by the element factor. With the matrices of OTU numbers and relative abundance of each OTU against relative abundance of elements and opportunistic pathogen densities by qPCR in water and sediment samples, it showed significant relationships between the microbial community structures and the densities of *Mycobacterium* spp., and two functional *Legionella pneumophila* genes (i.e., *sidF* and *rtxA*, *p* = 0.05) (Figure 3a). At *p* = 0.1 level, the community composition was also related positively with the densities of *Legionella* (Figure 3b). The vector direction of Zn was in opposite direction of other abiotic parameters including Al, K, Mn and Si, as well as biotic factors including concentrations of *V. vermiformis*, and *sidF,* and *rtxA* genes (Figure 4). 

## 4. Discussion

### 4.1. OPs in the Samples of Tank Water and Sediments

In this study the occurrences and densities of OPs were investigated and their associations with element concentrations was examined. The occurrences of *Mycobacterium* spp., *Legionella* spp., and *L. pneumophila* detected in this study were comparable to previous studies that investigated storage tank sediments [4] and drinking water distribution system [2]. In addition, higher occurrences of amoebae were also detected in the samples of sediment than those of water detected (Table 1). The free living amoebae may be beneficial to OPs [7]. *Acanthamoeba*, *V. vermiformis,* and other amoebae can serve as hosts for amoeba-resisting bacteria (ARB) like *Legionella* and *Mycobacterium* spp. [37,38]. The higher occurrences of amoebae in sediments than in water for the same storage tank suggest that they are more tightly bound to sediments and could be considered a reservoir of OPs. The targeted two genes (*rtxA* [39] and *sidF* [40]) encoding toxins can reflect virulence of *L. pneumophila*. Their co-occurrences and similar level of quantities in D50 water and D53 and D54 sediment samples, which belong to a Texas metropolitan area, indicated the presence of *L. pneumophila*. It has been reported that the growth of *Legionella* is positively supported by FLA, biofilm, and algae [41,42,43]. Positive correlations between *V. vermiformis* and total *Legionella* have been shown for drinking water samples [2]. The co-occurrence of amoebae, *Legionella* spp., and *L. pneumophila* in the sediments, and their relatively higher densities in the sediment samples than in water, suggest that sediments can serve as reservoirs of OPs in storage tank water, and therefore can potentially be released into the distribution system. Taken together, our study reveals the importance of sediments as reservoirs for OPs which should be considered as an important risk factor when developing microbial risk assessment models for various drinking water systems, especially for *Mycobacterium* and *Legionella* spp. The results further stress the importance of maintaining a good disinfectant residual throughout the drinking water distribution system including storage tanks.

### 4.2. Microbial Communities and Their Relationship with Elements

Dominant OTUs reported in this study shared similarities with other reports focused on DWDS. *Pseudomonadaceae* (mainly *Pseudomonas* spp.) were ubiquitously present in DWDS and proposed as an indicator of potential regrowth [44]. Aerobic heterotrophs *Comamonadaceae* and/or *Sphingomonadaceae* were documented as dominant groups in biofilms [45,46], purified water [47], chlorine treated water [48], and household water tanks [49]. *Sinobacteraceae* (*Nevskia* spp.) were found dominant in biofilms [50]. Nevertheless, the shared microbial compositions between the water and sediment in the same location samples indicated the close associations and interactions between these two different matrices. The bacterial flora attached to sediment could re-enter the water matrix due to normal activity or water flow. 

Both the qPCR results of *Mycobacterium* spp. and *Legionella* spp. were significantly associated with the microbial community composition. D39 water samples in OH had the second highest amount of *Mycobacterium* spp. It should be noticed that relative abundance of *Mycobacterium* spp. in microbial community was less than 1%, although it had high prevalence by qPCR. That community had a single dominant family of *Comamonadaceae*, which accounted for 75% of the total OTUs. D39 water samples also had the third highest amount of *Legionella* spp. of all the groups. The highest densities of *Mycobacterium* spp. and *Legionella* spp. were observed in D54 from Texas. The community featured with a couple of dominant families, with *Nitrospiraceae* accounting for 18.6% of total OTUs. 

The role that elements play in the microbial community structure of drinking water systems has been poorly documented. In our study we showed that for the sediment samples, elements such as Al, K, Mn and Si (Figure 4), as well as *V. vermiformis* and *L. pneumophila* detected by *sidF* and *rtxA* genes, correlated with the community structures in a similar way, which was the opposite with the heavy metal Zn. That may indirectly suggest which elements could be beneficial or harmful to *Legionella*. Additionally, cluster analysis revealed there was a high association of microbial community structure dominated with *Pseudomonas* spp. (33.5%) and Al and K in D54 (Figure 4), which, in part, is in agreement with previous findings on *Pseudomonas* spp. tolerance to aluminum [51,52]. Interestingly, while Al (III) and S could be reduced simultaneously by *Desulfovibrio* [53], *Desulfovibrio* was very scarce (<0.1%) in total OTUs. The unique community structure dominated by *Geothrix* (belonging to family *Holophagaceae*) (18.0%) and *Sulfuritalea* (17%) significantly associated with the highest iron (III) (50.0%) in D50, which is not surprising as these are known as Fe^+3^ reducing bacteria [54]. Freshwater sediments often contain large quantities of iron and manganese that undergo rapid cycling between reduced and oxidized states [17]. The cycling of these metals is driven by diffusion of soluble Mn^2+^ and Fe^2+^ into oxygenated environments, followed by oxidation and sedimentation of solid Mn^4+^ and Fe^3+^ elements to reducing regions. Microbial communities are thought to play a significant role in both the oxidation and reduction of manganese and iron [55,56]. 

It was well documented that magnesium had the potential to function as antibiotics to inhibit the growth of *P. aeruginosa* [20] and that iron is a key nutrient for pseudomonads [19]. That may explain the relationships between the qPCR results of *P. aeruginosa* and relative concentrations of magnesium and iron in sediments. On the other hand, since *V. vermiformis* is a potential host of *Legionella* spp. and *Mycobacterium* spp. [2], it is also possible that *P. aeruginosa* was hosted by *V. vermiformis*. Similarly, the associations of element Al with the quantities of *L. pneumophila,* representative of two genes *rtxA* and *sidF* in the sediments, also reflected the impact of metal on them. According to a previous study [57,58], the growth of *L. pneumophila* requires certain amounts of magnesium, cobalt, copper, iron, manganese, molybdenum, vanadium, or zinc to stimulate growth, while higher level of trace metal would inhibit the growth of *L. pneumophila* [59]. However, higher level of trace metal requirement was found for the growth of *P. aeruginosa* and its growth was stimulated by only four metals: calcium, iron, magnesium, and zinc [58].

## Figures and Tables

**Table 1:** The quantity of opportunistic pathogens in sediments and water of storage tanks using qPCR in two replicates.

Matrix	Location ID	*Acanthamoeba* spp.	*Vermamoeba vermiformis*	*Mycobacterium* spp.	*Pseudomonas aeruginosa*	*Legionella* spp.	*L. pneumophila* (*rtxA*)	*L. pneumophila* (*sidF*)
Sediment (GN G^−1^: genome copy number per gram sediment)	D39S	5 ± 1	0	(2.73 ± 0.68) × 10^4^	0	335 ± 31	0	0
	D40S	1 ± 1	4	(8.10 ± 1.16) × 10^3^	0	923 ± 34	0	0
	D41S	28 ± 7	0	(8.00 ± 1.20) × 10^1^	0	0	0	0
	D42S	0	0	(8.47 ± 10.04) × 10^3^	0	0	0	0
	D43S	0	0	0	0	0	0	0
	D50S	0	14 ± 22	(2.00 ± 4.00) × 10^1^	0	1 ± 3	0	0
	D53S	7	99 ± 60	(2.39 ± 2.24) × 10^3^	0	282 ± 113	25 ± 51	85 ± 69
	D54S	3	120 ± 43	(2.57 ± 0.88) × 10^5^	0	(7.20 ± 3.31) × 10^4^	300 ± 38	173 ± 87
	Average	6	29	3.79 × 10^4^	0	9.19 × 10^3^	41	32
	Occurrence	63%	50%	88%	0	50%	25%	25%
Water (GN L^−1^: genome copy number per liter water)	D39w	0	0	(2.33 ± 3.28) × 10^5^	70 ± 91	(1.24 ± 1.70) × 10^4^	0	0
	D40w	0	0	(5.63 ± 7.74) × 10^4^	0	(6.93 ± 9.76) × 10^4^	0	0
	D41w	50	0	6.91 × 10^3^	0	0	0	0
	D42w	0	0	3.37 × 10^4^	0	0	0	0
	D43w	0	0	2.52 × 10^3^	0	1.50 × 10^3^	0	0
	D50w	0	480 ± 670	(5.07 ± 0.03)×10^3^	2770 ± 14	(1.84 ± 1.46) × 10^4^	82 ± 117	36 ± 52
	D53w	0	0	0	83 ± 117	0	0	0
	D54w	0	98 ± 136	(2.68 ± 2.84) × 10^3^	371 ± 150	0	0	0
	Average	6	613	4.25 × 10^4^	412	1.27 × 10^4^	10	4
	Occurrence	13%	25%	88%	50%	50%	13%	13%

**Table 2:** Pearson’s linear correlation coefficients (R^2^) of opportunistic pathogens (OPs) with sediment elements in significant level 95% (*p* < 0.05).

Elements	Pathogens											
	*Acanthamoeba*		*Vermamoeba vermiformis*		*Mycobacterium*		*Legionella* spp.		*L. pneumophila* (*rtxA*)		*L. pneumophila* (*sidF*)	
	R^2^	P	R^2^	P	R^2^	P	R^2^	P	R^2^	P	R^2^	P
Al					0.93	<0.001	0.93	<0.001	0.92	<0.001	0.80	0.017
												
K					0.75	<0.031	0.77	<0.025	0.75	<0.031		

## Supplementary Materials

The following are available online at www.mdpi.com/ 2076-0817/6/4/54/s1, Table S1: Oligonucleotide taqMan qPCR primer sequences used to screen pathogens and human-fecal indicator *Bacteroidetes* and PCR primer sequences for clone sequencing, Table S2: Element compositions of storage tank sediments, Table S3: Element components in sediments of different locations, Table S4: major bacterial compositions with the relative abundance of OTUs >1% in any samples at 97% similarity level).
