# Supplementary material for: Opportunistic Pathogens and Microbial Communities and Their Associations with Sediment Physical Parameters in Drinking Water Storage Tank Sediments
Source: Pathogens. 2017 Oct 26;6(4):54. doi: 10.3390/pathogens6040054 (PMC5715195; doi:10.3390/pathogens6040054)
Supplement: Supplementary file 1 [file pathogens-06-00054-s001.pdf]

Table S1. Oligonucleotide taqMan qPCR primer sequences used to screen pathogens and human-fecal indicator *Bacteroidetes* and PCR primer sequences for clone sequencing

Target	Oligo name	Sequences (5'-3')	Tm (°C)	Detection limit (CE per reaction)	Reference
Legionella spp. (qPCR)	Leg F1c	TAGTGGAATTTCCGGTGTA	50	2	This study
	Leg R1c	CCAACAGCTAGTTGACATC			
	Leg-probe	6Fam-CGGCTACCTGGCCTAATACTGA-Tamra			
L. pneumophila (qPCR)	mipF1a	AGGATAAGTTGTCTTATAGCA	60	2	This study
	mipR1	TTAAGAACGTCTTTCA TTTG			
	mipProbe	6FAM-TAATCCGGAAGCAATGGCTAA-Tamra			
L. pneumophila (CYBR-Green qPCR)	rtxAf	ATTGCGCCTGGCAAACTTTAGGTG	64	2	Lu, et al. 2013
	rtxAr	GGCGCAAATCGTTTCCACCTTGTA			
L. pneumophila (CYBR-Green qPCR)	sidFf	ATTGTTCGCGAGGGTATGAAAGC G	64	2	Lu, et al. 2013
	sidFr	TCTTTCCAAGACAGACTCTCGCGT			
Mycobacterium spp. (qPCR)	23Smyco F	GGG GTGTGGTGTTTGAG	62	1	Bruijnesteijn van Coppenraet, 2004
	23Smyco R	CTCCCACGTCCTTCATC			
	23Smyco Probe	6-carboxyfluorescein-TGGATAGTGGTTGCGAGCATC-BHQ1			
Acanthamoeba spp. (qPCR)	TaqAcF1	CGACCAGCGATTAGGAGACG	60	2	Riviere et al. 2006
	TaqAcR1	CCGACGCCAAGGACGAC			
	TaqAcP1	FAM-TGAATACAAAACACCACCATCGGC GC-TAMRA			
Vermamoeba vermiformis (qPCR)	Hv1227F	TTA CGA GGT CAG GAC ACT GT	56	2	Kuiper et al. 2006
	Hv1728R	GAC CAT CCG GAG TTC TCG			

Table S2. Element compositions of storage tank sediments

Common Element	Occurrence	Average	Standard Deviation	Minimum Result	Median Result	Maximum Result
Al (wt%)	87.5%	3.35	3.26	0.01	2.00	10.43
Ca (wt%)	87.5%	3.17	2.39	0.59	2.05	6.50
Cl (wt%)	25%	0.96	0.04	0.93	0.96	0.98
Fe (wt%)	100%	15.96	15.52	1.36	13.31	49.98
K (wt%)	25%	0.77	0.16	0.65	0.77	0.88
Mg (wt%)	37.5%	0.61	0.08	0.54	0.65	0.65
Mn (wt%)	37.5%	3.63	1.84	1.62	4.04	5.22
Na (wt%)	62.5%	2.13	1.30	0.96	1.65	4.05
O (wt%)	100%	24.09	4.83	17.72	23.89	33.39
Si (wt%)	100%	7.07	5.94	2.13	5.21	20.60
Zn (wt%)	75%	18.71	14.91	3.01	17.53	41.54

Table S3. Element components in sediments of different locations

Sample	Coefficient of Uniformity	Coefficient of Curvature	Bulk Density	Particle Density	Porosity	Al (wt%)	Ca (wt%)	Cl (wt%)	Fe (wt%)	K (wt%)	Mg (wt%)	Mn (wt%)	Na (wt%)	O (wt%)	Si (wt%)	Zn (wt%)
D39	3.35	0.64	0.54	2.68	0.80	2.26	2.05		19.93					23.47	5.42	19.35
D40	4.33	0.57	0.51	3.58	0.86	2.31	4.33		19.54		0.65			22.86	5	15.71
D41	5.75	0.46	0.75	2.26	0.67	1.17	5.93		1.36					17.72	2.13	41.54
D42	4.37	0.61	0.70	1.39	0.50	1.74	6.5		3.72		0.65		1.65	18.92	3.93	29.03
D43	2.53	0.81				4.12	1.42	0.98	17.53	0.65	0.52	4.04	2.84	24.30	7.94	0.41
D50	5.56	1.33	1.02	1.22	0.17		0.59		49.98				1.13	26.44	2.71	3.62
D53	9.39	0.46	0.45	1.17	0.62	1.45	1.4	0.93	6.56			5.22	4.05	33.39	20.6	3.01
D54	2.93	0.74	0.83	1.24	0.33	10.43			9.08	0.88		1.62	5.96	25.58	8.86	0.03

Table S4. major bacterial compositions with the relative abundance of OTUs > 1% in any samples at 97% similarity level)

Sample Name	1st abundant	Percentage	2nd abundant	Percentage	Correlation (R^2)
D39 Water	Unclassified Comamonadaceae	73.7	Unclassified Sphingomonadaceae	6.3	0.69
D39 Sediment	Unclassified Sphingomonadaceae	23.1	Unclassified Comamonadaceae	20.8	
D40 Water	Unclassified Comamonadaceae	23.7	Reyranella massiliensis	6.4	0.47
D40 Sediment	Unclassified Syntrophobacteraceae	12.2	Unclassified Solibacterales	7.7	
D41 Water	Pseudomonas veronii	34.2	Unclassified Comamonadaceae	16.0	0.88
D41 Sediment	Pseudomonas veronii	49.4	Unclassified Massilia	17.4	
D42 Water	Pseudomonas veronii	46.5	Unclassified Massilia	14.9	0.98
D42 Sediment	Pseudomonas veronii	37.7	Unclassified Massilia	7.7	
D43 Water	Unclassified Pseudomonas	21.5	Unclassified MLE1-12	10.1	n/a
D43 Sediment	n/a				
D50 Water	Unclassified Novosphingobium	26.1	Unclassified Comamonadaceae	6.4	0.38
D50 Sediment	Unclassified Geothrix	18.0	Unclassified Sulfuritalea	17.4	
D51 Water	Unclassified Acinetobacter	49.6	Unclassified Pseudomonadaceae	7.0	
D51 Sediment	Sphingomonas wittichii	38.0	Unclassified Comamonadaceae	18.3	
D53 Water	Blastomonas natatoria	26.5	Unclassified Acinetobacter	11.9	0.11
D53 Sediment	unclassified PK29	9.2	unclassified s-70	8.6	
D54 Water	Unclassified Pseudomonas	48.9	Unclassified Comamonadaceae	6.8	0.07
D54 Sediment	Unclassified Nitrospira	15.7	Unclassified Gemm-1	13.1	
D55 Water	Nevskia ramosa	93.4	Unclassified Sphingomonadaceae	2.0	
D55 Sediment	Unclassified Sphingomonadaceae	42.1	Unclassified Comamonadaceae	24.2	
D56 Water	Unclassfied Enterobacteriaceae	17.2	Unclassified Pseudomonas	6.3	
D56 Sediment	Sphingomonas asaccharolytica	15.8	Dongia mobilis	8.1	
D60 Sediment	Unclassified Novosphingobium	33.0	Peredibacter starrii	19.9	
